# Lung cancer and altered brain structure: A causal inference based on MR

**DOI:** 10.1097/MD.0000000000050698

**Published:** 2026-09-25

**Authors:** Zhen Yuan, Zhiwei Xu, Shuyue Lv, Yin Dong, Aoli Chen, Wenchao Wang, Jiwei Cheng

**Affiliations:** a Department of Neurology, Putuo Hospital, Shanghai University of Traditional Chinese Medicine, Shanghai, China; b School of Acupuncture and Tuina, Shandong University of Traditional Chinese Medicine, Jinan, Shandong, China; c Department of Cardiology, Putuo Hospital, Shanghai University of Traditional Chinese Medicine, Shanghai, China; d Department of Community Health Service, Changfeng Community Health Center, Putuo District, Shanghai, China.

**Keywords:** brain morphology, cortical structure, lung cancer, Mendelian Randomization, psychiatric comorbidity

## Abstract

Lung cancer is one of the most prevalent malignancies globally and imposes a substantial burden on mortality and healthcare costs. Emerging clinical evidence suggests that lung cancer may influence brain morphology, particularly the cerebral cortex. This study employed Mendelian Randomization (MR) to investigate the potential causal relationship between lung cancer and cortical structural alterations. Single nucleotide polymorphisms associated with small cell lung cancer (SCLC) and non-SCLC (NSCLC) were instrumental variables derived from the FinnGen consortium. Summary-level data on brain cortical structures were obtained from the Enhancing Neuroimaging Genetics through Meta-Analysis consortium. MR analyses were conducted using inverse variance weighted regression, MR-Egger regression, and weighted median methods. SCLC was associated with a reduced surface area of the rostral anterior cingulate cortex (*β* = −0.038, 95% confidence interval [CI]: −0.057–−0.019; false discovery rate-corrected *P* = .007). For NSCLC, we observed nominal associations with decreased cortical thickness in the paracentral (*β* = −0.032, 95% CI: −0.056–−0.008; *P* = .008) and transverse temporal regions (*β* = −0.032, 95% CI: −0.056–−0.008; *P* = .009), and an increased surface area of the lingual gyrus (*β* = 0.028, 95% CI: 0.002–0.054; *P* = .034). Sensitivity analyses showed no evidence of directional pleiotropy, and leave-one-out tests indicated that results were not driven by single instruments. Mediation analyses provided no support for indirect effects via insomnia, anxiety, or depression. Our findings support a causal link between SCLC and reduced rostral anterior cingulate cortex surface area and suggest additional NSCLC-related cortical alterations in sensorimotor, auditory, and visual regions. These results highlight potential neuropsychiatric and cognitive implications in lung cancer and warrant further mechanistic investigation.

## 1. Introduction

Lung cancer is one of the most prevalent malignancies worldwide and is characterized by high incidence and mortality rates.^[1]^ Based on histopathological features, lung cancer is broadly classified into 2 major subtypes: small cell lung cancer (SCLC) and non-SCLC (NSCLC).^[2]^ With the increasing number of smokers^[3]^ and exposure to air pollution,^[4]^ the global burden of lung cancer continues to grow. In 2020 alone, there were an estimated 2.2 million new cases and 1.8 million deaths attributed to lung cancer worldwide.^[5]^ Given this alarming trend, urgent public health strategies are required for effective prevention and treatment.

Recent research has suggested that lung cancer may be associated with alterations in the structure of the cerebral cortex. Clinical neuroimaging studies have shown that even in the absence of brain metastasis, lung cancer patients may exhibit increased cortical thickness in regions such as the left insular gyrus and central sulcus during early disease stages.^[6]^ However, these findings are primarily based on observational data, which are vulnerable to confounding and reverse causation, thereby limiting causal interpretability. Mendelian Randomization (MR) offers a robust alternative by using genetic variants as instrumental variables (IVs) to infer causality and minimize bias.^[7]^

Insomnia is a common comorbidity among patients with lung cancer. Evidence suggests that individuals with lung cancer report poorer sleep quality than those with other malignancies or the general population.^[8]^ Sleep deprivation has also been associated with reduced thalamic gray matter volume.^[9]^ According to the “lung-brain axis” hypothesis, pulmonary diseases may disrupt circadian rhythm perception and influence mental health, potentially contributing to anxiety and depression.^[10]^ To explore whether these psychiatric factors mediate the relationship between lung cancer and cortical structural changes, we incorporated genome-wide association study (GWAS) data on insomnia, anxiety, and depression into a Mendelian mediation analysis.

In this study, we leveraged summary statistics from a large-scale GWAS of brain cortical structures based on magnetic resonance imaging (MRI) to examine the causal relationships between both SCLC and NSCLC and alterations in cortical morphology. Furthermore, we applied a mediation MR analysis to investigate the potential intermediary roles of insomnia, anxiety, and depression in the association between lung cancer and brain structure.

## 2. Methods

### 2.1. Core assumptions of MR

The analytical framework of this study is shown in Figure 1. MR relies on 3 core assumptions to ensure a valid causal inference^[11]^:

**Figure 1.**
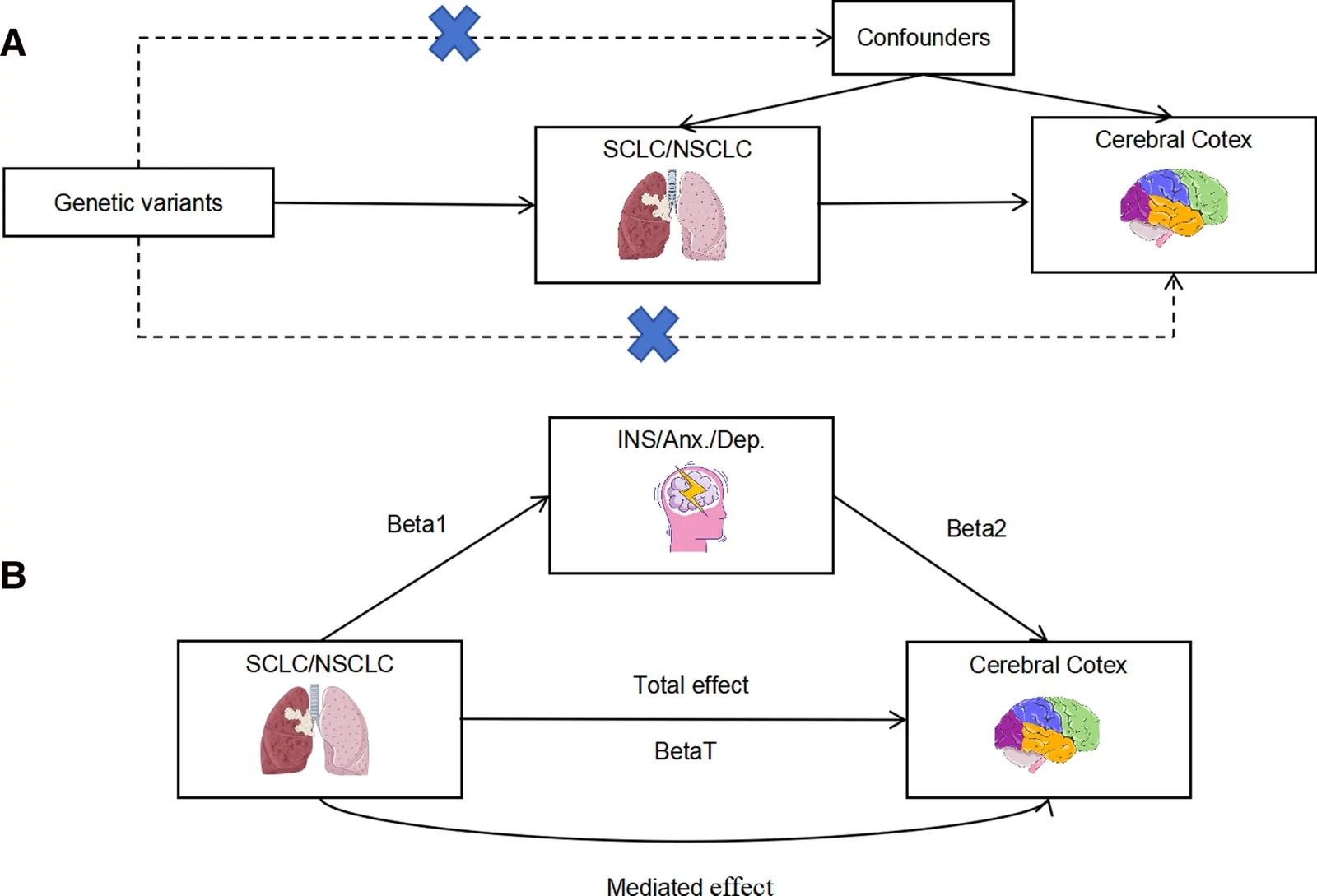
Flowchart of MR analysis. (A) Univariable MR analysis flow chart. (B) Mediation MR analysis flow chart. Anx = anxiety, Dep = depression, INS = insomnia, MR = Mendelian randomization, NSCLC = non-small cell lung cancer, SCLC = small cell lung cancer. The brain/cerebral cortex and lung graphical elements were adapted from Servier Medical Art (https://smart.servier.com/), licensed under CC BY 4.0. The epilepsy/seizure profile illustration was obtained from Vecteezy (Idalba Granada): https://www.vecteezy.com/png/53135204-epilepsy-seizure-profile, and is used with the required attribution. The overall figure was created and arranged by the authors.

1. Relevance assumption: IVs must be strongly associated with exposure of interest. This is typically confirmed using a genome-wide significance threshold, often defined as a *P* value < 1 × 10^−5^.
2. Independence assumption: The IVs must be independent of confounding factors that could bias the association between exposure and outcome. This ensures that external variables do not drive the observed relationships.
3. Exclusion restriction assumption: The IVs must influence the outcome exclusively through exposure, not through alternative biological pathways or pleiotropic effects; when these 3 assumptions are satisfied, MR analysis can effectively mitigate the impact of unmeasured confounding and reverse causation, thereby enhancing the credibility of causal inference.

### 2.2. Data sources

All GWAS summary statistics used in this study were obtained from publicly available databases. The GWAS summary data for SCLC and NSCLC were sourced from the FinnGen consortium, a collaborative project between Finnish biobanks and the National Healthcare Registry. As of August 2020, FinnGen has included genetic and clinical data from approximately 412,000 participants collected across 9 biobanks, research institutes, hospitals, international pharmaceutical partners, and Finnish Biobank Cooperative.^[12]^ The GWAS dataset for SCLC included 717 cases and 314,193 controls, while the NSCLC dataset included 5315 cases and 314,193 controls. Summary statistics for cortical brain structure were obtained from the Enhancing NeuroImaging Genetics through Meta-Analysis consortium.^[13]^ This dataset was based on a large-scale GWAS meta-analysis of MRI data from 51,665 individuals. Cortical thickness and surface area were measured in 34 distinct brain regions as well as across the entire cortex, based on the Desikan–Killiany atlas. Measurements were performed across the whole brain for statistical estimation.^[14]^

Importantly, cortical thickness (in millimeters) and surface area (in square millimeters) from Enhancing Neuroimaging Genetics through Meta-Analysis were analyzed in their original MRI-derived physical units. These phenotypes were not z-standardized in the GWAS summary statistics. Therefore, MR effect estimates (*β*) should be interpreted as absolute differences in cortical metrics (mm or mm^2^) per unit increase in genetic liability to lung cancer.

To reduce the risk of sample overlap, we selected summary-level GWAS data from the United Kingdom Biobank (UKB) for traits that potentially mediate the relationship between lung cancer and brain structure. The UKB is a large-scale biomedical resource that collects genetic and health-related data from over 500,000 participants in the United Kingdom.^[15]^ Specifically, the GWAS data for insomnia included 43,474 cases and 13,741 controls, anxiety included 463 cases and 455,885 controls, and depression included 376 cases and 455,972 controls. Detailed information on all the datasets is provided in Tables S1 and S2, Supplemental Digital Content 1.

### 2.3. Selection of genetic IVs

To satisfy the relevance assumption of MR, we selected single nucleotide polymorphisms (SNPs) that were strongly associated with lung cancer and cerebral cortical structure using a significance threshold of *P* < 1 × 10^−5^. Similarly, for potential mediators, such as insomnia, anxiety, and depression, SNPs associated with the same threshold (*P* < 1 × 10^−5^) were extracted. To ensure independence among the IVs, we performed linkage disequilibrium clumping using the European ancestry reference panel. SNPs were pruned based on an *R*^2^ < 0.001 within a 10,000-kilobase (kb) window. After linkage disequilibrium pruning, we calculated the *F*-statistic for each IV to assess instrument strength. SNPs with *F*-values > 10 were retained to reduce the risk of weak instrument bias.^[16]^

To minimize the influence of potential confounding factors, we excluded SNPs associated with traits known to be linked to both lung cancer and brain structure. Based on previous literature, educational attainment, body mass index, smoking behavior, and kidney dysfunction are potential confounders of this relationship.^[17–22]^ Therefore, we used the PhenoScanner database (v2.0) via the R package PhenoScanner (v1.0) to identify and exclude SNPs that were significantly associated with these traits.

### 2.4. Statistical analysis

We applied 3 MR methods to evaluate causal effects: inverse variance weighted (IVW), which served as the primary analysis^[23]^; weighted median; and MR-Egger regression, both used for sensitivity analyses to complement the IVW approach.^[24,25]^ The IVW method assumes that all IVs are valid. By contrast, the weighted median method provides robust causal estimates even when up to 50% of the instruments are invalid, and the MR-Egger method allows for directional pleiotropy by adjusting for unbalanced horizontal effects. The results are presented as beta coefficients (*β*) with corresponding 95% confidence intervals (CIs), quantifying the magnitude and direction of the causal association between exposure and outcome. We performed MR Pleiotropy RESidual Sum and Outlier analysis to detect and iteratively remove outlier SNPs, thus reducing the potential impact of horizontal pleiotropy. Heterogeneity among genetic instruments was assessed using Cochran *Q* test, and directional pleiotropy was evaluated using the intercept term from the MR-Egger regression.^[26]^

For the mediation MR analysis, we first estimated the total causal effect (*β*_t_) of lung cancer on the cortical structure. We then computed the effect of lung cancer on each mediator (*β*_1_) and the effect of the mediator on the cortical structure (*β*_2_). The indirect effect (mediation effect) was calculated using the product-of-coefficients method as *β*_1_ × *β*_2_, and the mediation proportion was determined as (*β*_1_ × *β*_2_)/ *β*_t_.^[26]^ To account for multiple testing, we applied a false discovery rate (FDR) correction to the IVW results, with statistical significance defined as FDR < 0.05.^[27]^ A causal relationship was considered statistically significant only when the FDR-corrected *P* value was < .05, and no significant heterogeneity, pleiotropy, or reverse causation was observed. Associations with uncorrected *P* values < .05 but without passing FDR correction were considered nominally significant, and all statistical analyses were conducted using R software (version 4.1.1; R Foundation for Statistical Computing, Vienna, Austria) in RStudio (Posit, PBC/Posit, Boston).

## 3. Results

### 3.1. Selection of genetic instruments

In the univariable MR analysis, we identified 13 SNPs that were significantly associated with SCLC and an additional 37 SNPs that were associated with NSCLC to assess their causal effects on cortical structures. For the reverse MR analysis, we utilized 12 to 63 SNPs representing the surface area and thickness of the entire cerebral cortex and its subregions. In the mediation MR analysis, 28 SNPs were used to represent insomnia, 20 SNPs to represent depression, and 21 SNPs to represent anxiety as potential mediators. Detailed information on the selected SNPs is provided in Table S3, Supplemental Digital Content 2.

### 3.2. Causal effects of lung cancer on cortical structure

We observed several statistically and nominally significant causal associations between lung cancer and cortical measures (Table 1). SCLC was associated with a significant reduction in the surface area of the rostral anterior cingulate cortex (ACC, rACC) (*β* = –0.038, 95% CI:−0.057–−0.019, P.FDR = 0.007). SCLC was nominally associated with a decrease in the cortical thickness of the pars triangularis (*β* = −0.032, 95% CI: −0.056–−0.008, *P* = .008) and an increase in the thickness of the rACC (*β* = 0.027, 95% CI: 0.002–0.051, *P* = .032).

**Table 1:** All significant univariate MR results.

Exposure	Outcome	Method	Beta	95%CI	*P* value
SCLC	SA of the rACC	IVW	0.038	(−0.057–−0.019)	.0001
		MR-Egger	−0.029	(−0.106–0.049)	.481
		Weighted median	−0.039	(−0.064–−0.014)	.003
	TH of the PT	IVW	−0.032	(−0.041–−0.003)	.024
		MR-Egger	−0.0006	(−0.059–0.057)	.984
		Weighted median	−0.028	(−0.055–−0.001)	.044
	TH of the rACC	IVW	0.027	(0.002–0.051)	.032
		MR-Egger	−0.032	(−0.101–0.036)	.376
		Weighted median	0.034	(0.004–0.063)	.024
NSCLC	TH of the PC	IVW	−0.032	(−0.056–−0.008)	.008
		MR-Egger	−0.017	(−0.069–0.034)	.518
		Weighted median	−0.024	(−0.062–0.013)	.202
	TH of the TT	IVW	−0.032	(−0.056–−0.008)	.009
		MR-Egger	−0.014	(−0.066–0.039)	.611
		Weighted median	−0.035	(−0.071–0.002)	.064
	SA of the LG	IVW	0.028	(0.002–0.054)	.034
		MR-Egger	0.033	(−0.025–0.09)	.271
		Weighted median	0.023	(−0.014–0.06)	.229

IVW = inverse variance weighted, LG = lingual gyrus, MR = Mendelian randomization, NSCLC = non-small cell lung cancer, PC = paracentral lobule, PT = pars triangularis, rACC = rostral anterior cingulate cortex, SA = surface, SCLC = small cell lung cancer, TH = thickness, TT = transverse temporal gyrus.

For NSCLC, significant associations were found with decreased cortical thickness in the paracentral (*β* = −0.032, 95% CI: −0.056–−0.008, *P* = .008) and transverse temporal regions (*β* = −0.032, 95% CI: −0.056–−0.008, *P* = .009) and an increased surface area of the lingual gyrus (*β* = 0.028, 95% CI: 0.002–0.054, *P* = .034). Additional details are provided in Table S4, Supplemental Digital Content 3. Funnel plots revealed no evidence of asymmetry, indicating a low risk of directional pleiotropy (Fig. S1, Supplemental Digital Content 4). Leave-one-out analysis further confirmed that the observed causal effects were not driven by any single instrumental SNP (Fig. S2, Supplemental Digital Content 5).

### 3.3. Causal effects of cerebral cortex on lung cancer

In reverse MR analysis assessing the causal effects of cortical structure on lung cancer, we identified several nominally significant associations. Increased medial orbitofrontal cortex thickness was associated with a reduced risk of SCLC (*β* = −0.914, 95% CI:−1.607–−0.221, *P* = .009). Additionally, a greater surface area of the superior temporal region was also associated with a lower risk of SCLC (*β* = −0.494, 95% CI:−0.930–−0.058, *P* = .0264).

For NSCLC, increased surface area of the lateral orbitofrontal cortex (*β* = −0.157, 95% CI:−0.292–−0.022, *P* = .023) and increased thickness of the superior temporal region (*β* = −0.178, 95% CI:−0.352–−0.005, *P* = .044) were both associated with a reduced incidence. Similarly, greater thickness of the entorhinal cortex was associated with a lower risk of NSCLC (*β* = −0.157, 95% CI:−0.292–−0.022, *P* = .023). However, the MR-Egger intercept test showed a significant *Q* test *P* value (*P* = .037), indicating possible horizontal pleiotropy among the IVs used in this model. Therefore, this finding should be interpreted with caution, as it may reflect a potential but not definitive association. Funnel plots demonstrated no apparent asymmetry, suggesting a low risk of directional pleiotropy (Fig. S3, Supplemental Digital Content 6). Leave-one-out sensitivity analyses indicated that the observed causal effects were not driven by any single instrumental SNP (Fig. S4, Supplemental Digital Content 7). The full results of the effects of the cortical structure on lung cancer are illustrated in Figure 2. Detailed results of the reverse MR analysis are presented in Table S5, Supplemental Digital Content 8.

**Figure 2.**
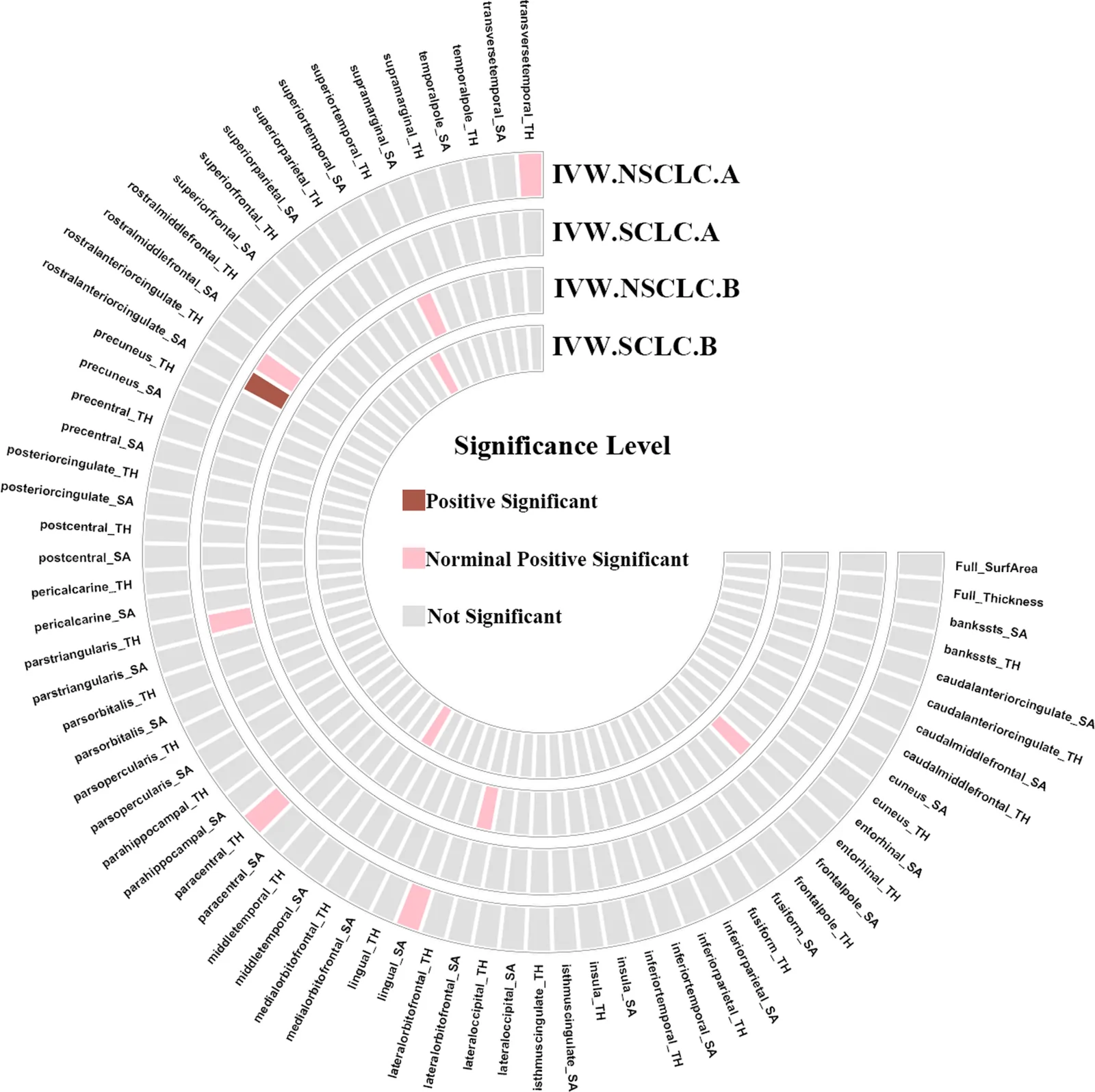
Circular heatmap of results showing the causal associations between lung cancer subtypes (NSCLC and SCLC) and cortical structures. IVW.NSCLC.A, causal effects of NSCLC on cerebral cortex; IVW.NSCLC.B, causal effects of cerebral cortex on NSCLC; IVW.SCLC.A, causal effects of SCLC on cerebral cortex; IVW.SCLC.B, causal effects of cerebral cortex on SCLC. IVW = inverse variance weighted, MR = Mendelian randomization, NSCLC = non-small cell lung cancer, SCLC = small cell lung cancer.

### 3.4. Mediating effects of insomnia, anxiety, and depression

To minimize the impact of sample overlap, we used GWAS summary data on insomnia, anxiety, and depression from the UKB as mediators in our MR mediation analysis. We performed mediation MR analyses on the significant and nominally significant associations previously identified between lung cancer and cortical structures. None of the 3 psychological traits (insomnia, anxiety, or depression) were found to mediate these associations. Detailed results of the mediation analysis are presented in Table 2.

**Table 2:** Results of mediation effect analysis.

Exposure	Mediate	Outcome	Total effect	Mediate effect	Mediating effect ratio (%)	*P* value
SCLC	Depression	rostralanteriorcingulate_surfavg	−0.038 (−0.057–−0.019)	−0.000104 (−0.001477–0.001269)	0.27	.88
SCLC	Anxiety	rostralanteriorcingulate_surfavg	−0.038 (−0.057–−0.019)	−0.000344 (−0.001709–0.001022)	0.90	.62
SCLC	Insomnia	rostralanteriorcingulate_surfavg	−0.038 (−0.057–−0.019)	−0.000106 (−0.001338–0.001127)	0.28	.87
SCLC	Depression	parstriangularis_thickavg	−0.022 (−0.041–−0.003)	0.000045 (−0.000562–0.000652)	−0.20	.88
SCLC	Anxiety	parstriangularis_thickavg	−0.022 (−0.041–−0.003)	−0.000402 (−0.001778–0.000975)	1.81	.57
SCLC	Insomnia	parstriangularis_thickavg	−0.022 (−0.041–−0.003)	−0.000715 (−0.002402–0.000971)	3.23	.41
SCLC	Depression	rostralanteriorcingulate_thickavg	0.027 (0.002–0.051)	−0.000070 (−0.000999–0.000859)	−0.26	.88
SCLC	Anxiety	rostralanteriorcingulate_thickavg	0.027 (0.002–0.051)	−0.000315 (−0.001573–0.000944)	−1.17	.62
SCLC	Insomnia	rostralanteriorcingulate_thickavg	0.027 (0.002–0.051)	0.000161 (−0.001091–0.001414)	0.60	.80
NSCLC	Depression	paracentral_thickavg	−0.032 (−0.056–−0.008)	0.000076 (−0.001793–0.001946)	−0.24	.94
NSCLC	Anxiety	paracentral_thickavg	−0.032 (−0.056–−0.008)	0.000361 (−0.002383–0.003106)	−1.12	.80
NSCLC	Insomnia	paracentral_thickavg	−0.032 (−0.056–−0.008)	−0.000321 (−0.001489–0.000846)	1.00	.59
NSCLC	Depression	transversetemporal_thickavg	−0.032 (−0.041–−0.003)	−0.000006 (−0.000182–0.000169)	0.02	.94
NSCLC	Anxiety	transversetemporal_thickavg	−0.032 (−0.041–−0.003)	−0.001981 (−0.004812–0.000850)	6.18	.17
NSCLC	Insomnia	transversetemporal_thickavg	−0.032 (−0.041–−0.003)	−0.000317 (−0.001472–0.000839)	0.99	.59
NSCLC	Depression	lingual_surfavg	0.027 (0.002–0.054)	0.001188 (−0.001551–0.003926)	4.42	.40
NSCLC	Anxiety	lingual_surfavg	0.027 (0.002–0.054)	0.001188 (−0.001551–0.003926)	4.42	.40
NSCLC	Insomnia	lingual_surfavg	0.027 (0.002–0.054)	−0.000301 (−0.001503–0.000900)	−1.12	.62

NSCLC = non-small cell lung cancer, SCLC = small cell lung cancer.

## 4. Discussion

In this study, we comprehensively analyzed the association between NSCLC and SCLC both cortical thickness and surface area across various brain regions. Notably, we identified a significant relationship between SCLC and reduced surface area in the rACC, which is consistent with previous clinical findings.^[28]^ This reduction may reflect a loss of neurons and dendritic complexity in the rACC, potentially contributing to symptoms such as apathy, diminished self-awareness, motor neglect, and impaired pain perception.^[29]^ The rACC, a subregion of the ACC, plays a pivotal role in emotional regulation and is implicated in major depressive disorders.^[30]^ Epidemiological studies have shown that lung cancer patients are at an elevated risk for depression compared to those with other cancer types,^[31,32]^ with 1 study reporting a threefold higher incidence of depression in patients with SCLC than in those with NSCLC (*P* < .0001).^[33]^ These findings support our results and suggest a potential neural basis for increased vulnerability to affective disorders in this population.

Interestingly, SCLC was also nominally associated with increased cortical thickness in the rACC region. This apparent contradiction (a reduction in surface area but an increase in thickness) may be due to distinct neurobiological mechanisms. The cortical surface area is largely determined by genetic regulation of neuronal progenitor cell proliferation during early development, whereas cortical thickness is influenced more by gene expression and epigenetic regulation in the adult brain.^[22]^ An alternative hypothesis is that the observed increase in cortical thickness may reflect an acute or subacute neuroinflammatory response, including glial activation and local edema, while chronic neuronal loss may ultimately reduce the surface area over time.^[34]^ Further mechanistic studies are required to elucidate these processes. In NSCLC, we observed a nominal association with an increased surface area of the lingual gyrus, a region implicated in visual processing. This finding aligns with previous clinical studies reporting altered lingual gyrus morphology in lung cancer patients.^[6]^ Although a reduction in lingual gray matter volume has been associated with cognitive decline in breast cancer survivors,^[35]^ our observation of increased surface area may reflect compensatory adaptation. For instance, patients may develop enhanced visual memory or imagery processing to offset other cognitive deficits. SCLC is also associated with a reduced thickness of the pars triangularis, a component of Broca area responsible for language production.^[36]^ This structural change may underlie language difficulties observed in patients with advanced lung cancer,^[37]^ potentially contributing to social withdrawal and depressive symptoms. Additionally, NSCLC is associated with reduced cortical thickness in the paracentral lobule, a region essential for motor function. This could reflect disuse atrophy due to physical inactivity, cachexia, or other disease-related impairments.^[38]^ We also found changes in the transverse temporal gyrus, a key region of the auditory cortex, in patients with NSCLC. Although our findings suggest structural changes in auditory processing regions, there is currently a lack of clinical evidence linking lung cancer to hearing impairments. Further clinical investigation is required to validate this relationship.

Patients with SCLC often exhibit systemic inflammatory responses, during which pro-inflammatory cytokines can cross the blood-brain barrier and activate immune responses within the central nervous system.^[34]^ Tumor-associated inflammation may directly damage neural tissues in the ACC. Previous research has shown that in patients with severe depression, the levels of quinolinic acid, a neurotoxic metabolite, are significantly elevated in several ACC subregions, indicating that inflammatory metabolite accumulation within the ACC can lead to structural degeneration.^[39]^ Quinolinic acid is a potent N-methyl-D-aspartate receptor agonist, and elevated levels may result in excitotoxicity and lipid peroxidation, both of which contribute to neuronal injury.

In addition to humoral pathways, neuroimmune interactions may also mediate the effects of SCLC on the brain structure. Owing to its high immunogenicity, SCLC is frequently associated with paraneoplastic neurological syndromes, which are immune-mediated complications of malignancy.^[40]^ These syndromes occur when the immune system erroneously targets neural antigens, mistaking them for tumor antigens. A classic example is limbic encephalitis, which is observed in approximately 50% of SCLC patients.^[41]^ This condition involves tumor-associated autoantibodies and cytotoxic T-cell responses attacking the limbic structures, including the hippocampus, amygdala, and ACC. Clinically, this can manifest as personality changes, irritability, anxiety, and memory impairment, symptoms indicative of damage to emotion-related brain regions, such as the ACC. Furthermore, SCLC often produces ectopic hormones, such as adrenocorticotropic hormone, leading to chronic exposure to elevated glucocorticoid levels. Prolonged hypercortisolemia has been associated with gray matter atrophy in regions such as the hippocampus and ACC.^[42]^ Thus, endocrine dysregulation may further exacerbate the structural reductions in the ACC. Taken together, these findings suggest that systemic inflammation, immune-mediated neural injury, and endocrine abnormalities may synergistically contribute to the pronounced reduction in ACC surface area observed in patients with SCLC.

Recent MR studies have begun to explore cancer–brain interactions, further underscoring the novelty of our work. For example, a large MR analysis of breast cancer reported that genetically predicted estrogen receptor–negative breast cancer was causally associated with widespread cortical atrophy across frontal, parietal, and occipital regions, offering a genetic explanation for cancer-associated cognitive impairment.^[43]^ Similarly, bidirectional MR analyses have revealed associations between liver tumors and cortical surface area variation, suggesting that liver tumor pathology and brain morphology may be linked through a liver–brain axis.^[44]^ More recently, an MR study of ovarian cancer demonstrated 2-way causal links between brain imaging phenotypes and ovarian cancer risk, highlighting that alterations in cortical and subcortical structures may not only be a consequence of cancer but also serve as potential biomarkers for cancer susceptibility.^[45]^ Together, these studies emphasize that cancer may exert subtype-specific and system-wide effects on brain morphology. Our study expands this literature by providing the first MR evidence that lung cancer, particularly SCLC and NSCLC, can influence distinct cortical regions involved in emotion regulation, sensory processing, and motor function.

This article examines structural remodeling of the brain in patients with lung cancer, offering insights into the underlying neural mechanisms of emotional disorders and cognitive impairments commonly observed in this population. By integrating neuroscience perspectives, the findings support the potential need for targeted interventions, such as anti-inflammatory treatments and cognitive rehabilitation, to enhance the quality of life of lung cancer patients. This study has several strengths. First, we applied a bidirectional 2-sample MR approach, which improves causal inference by accounting for potential confounding factors and reverse causation. Second, we implemented rigorous criteria for SNP selection using strict thresholds for both *P* values and *F*-statistics to ensure robust IVs. Third, our sensitivity analyses did not reveal substantial horizontal pleiotropy, which further strengthened the validity of our findings. Nonetheless, this study had several limitations. GWAS summary statistics were derived from European ancestry populations, potentially limiting the generalizability of our results to other ethnic groups.

## Acknowledgments

The authors wish to acknowledge the participants and investigators of the FinnGen study, the UK Bank study, and the ENIGMA consortium for providing GWAS summary data.

## Author contributions

**Conceptualization:** Zhen Yuan, Zhiwei Xu, Wenchao Wang.

**Data curation:** Zhen Yuan, Yin Dong, Aoli Chen.

**Formal analysis:** Zhiwei Xu.

**Funding acquisition:** Jiwei Cheng.

**Investigation:** Aoli Chen.

**Methodology:** Zhen Yuan.

**Project administration:** Shuyue Lv.

**Software:** Yin Dong.

**Supervision:** Shuyue Lv, Jiwei Cheng.

**Validation:** Jiwei Cheng.

**Visualization:** Wenchao Wang

**Writing** – **original draft:** Zhen Yuan, Zhiwei Xu, Yin Dong, Aoli Chen, Wenchao Wang.

**Writing** – **review & editing:** Shuyue Lv, Jiwei Cheng.



















## References

[1] Forde PM Spicer J Lu S. Neoadjuvant nivolumab plus chemotherapy in resectable lung cancer. N Engl J Med. 2022;386:1973–85. 10.1056/NEJMoa2202170 PMC9844511 35403841

[2] Li Y Yan B He S. Advances and challenges in the treatment of lung cancer. Biomed Pharmacother. 2023;169:115891. 10.1016/j.biopha.2023.115891 37979378

[3] Zhou RX Liao HJ Hu JJ Xiong H Cai XY Ye DW. Global burden of lung cancer attributable to household fine particulate matter pollution in 204 countries and territories, 1990 to 2019. J Thorac Oncol. 2024;19:883–97. 10.1016/j.jtho.2024.01.014 38311022

[4] Xue Y Wang L Zhang Y Zhao Y Liu Y. Air pollution: a culprit of lung cancer. J Hazard Mater. 2022;434:128937. 10.1016/j.jhazmat.2022.128937 35452993

[5] Leiter A Veluswamy RR Wisnivesky JP. The global burden of lung cancer: current status and future trends. Nat Rev Clin Oncol. 2023;20:624–39. 10.1038/s41571-023-00798-3 37479810

[6] Zhang DF Ma H Yang GJ. Blood-brain barrier and brain structural changes in lung cancer patients with non-brain metastases. Front Oncol. 2022;12:1015011. 10.3389/fonc.2022.1015011 PMC9623018 36330467

[7] Ference BA Holmes MV Smith GD. Using mendelian randomization to improve the design of randomized trials. Cold Spring Harb Perspect Med. 2021;11:a040980. 10.1101/cshperspect.a040980 PMC8247560 33431510

[8] Dean GE Abu SE Yingrengreung S. Sleeping with the enemy: sleep and quality of life in patients with lung cancer. Cancer Nurs. 2015;38:60–70. 10.1097/NCC.0000000000000128 25486204

[9] Liu C Kong XZ Liu X Zhou R Wu B. Long-term total sleep deprivation reduces thalamic gray matter volume in healthy men. Neuroreport. 2014;25:320–3. 10.1097/WNR.0000000000000091 24346259

[10] Bajinka O Simbilyabo L Tan Y Jabang J Saleem SA. Lung-brain axis. Crit Rev Microbiol. 2022;48:257–69. 10.1080/1040841X.2021.1960483 34348558

[11] Zhang H. Pros and cons of Mendelian randomization. Fertil Steril. 2023;119:913–6. 10.1016/j.fertnstert.2023.03.029 PMC10234673 36990264

[12] Kurki MI Karjalainen J Palta P. FinnGen provides genetic insights from a well-phenotyped isolated population. Nature. 2023;613:508–18. 10.1038/s41586-022-05473-8 PMC9849126 36653562

[13] Grasby KL Jahanshad N Painter JN. The genetic architecture of the human cerebral cortex. Science. 2020;367:eaay6690. 10.1126/science.aay6690 PMC7295264 32193296

[14] Zhou S Wei T Liu X. Causal effects of COVID-19 on structural changes in specific brain regions: a Mendelian randomization study. BMC Med. 2023;21:261. 10.1186/s12916-023-02952-1 PMC10354891 37468885

[15] Rusk N. The UK Biobank. Nat Methods. 2018;15:1001. 10.1038/s41592-018-0245-2 30504882

[16] Yang Z He H He G Zeng C Hu Q. Investigating causal effects of hematologic traits on lung cancer: a mendelian randomization study. Cancer Epidemiol Biomarkers Prevent. 2024;33:96–105. 10.1158/1055-9965.EPI-23-0725 37909945

[17] Zhou H Zhang Y Liu J. Education and lung cancer: a Mendelian randomization study. Int J Epidemiol. 2019;48:743–50. 10.1093/ije/dyz121 31219597

[18] Farah MJ. Socioeconomic status and the brain: prospects for neuroscience-informed policy. Nat Rev Neurosci. 2018;19:428–38. 10.1038/s41583-018-0023-2 29867123

[19] Zhou W Liu G Hung RJ. Causal relationships between body mass index, smoking and lung cancer: univariable and multivariable Mendelian randomization. Int J Cancer. 2021;148:1077–86. 10.1002/ijc.33292 PMC7845289 32914876

[20] Westwater ML Vilar-Lopez R Ziauddeen H Verdejo-Garcia A Fletcher PC. Combined effects of age and BMI are related to altered cortical thickness in adolescence and adulthood. Dev Cogn Neurosci. 2019;40:100728. 10.1016/j.dcn.2019.100728 PMC6913515 31751856

[21] Qian H Li S Hu Z. Association between renal dysfunction and outcomes of lung cancer: a systematic review and meta‑analysis. Oncol Lett. 2024;28:514. 10.3892/ol.2024.14648 PMC11378011 39247494

[22] Chen X Kong J Pan J. Kidney damage causally affects the brain cortical structure: a Mendelian randomization study. Ebiomedicine. 2021;72:103592. 10.1016/j.ebiom.2021.103592 PMC8498227 34619639

[23] Wu F Huang Y Hu J Shao Z. Mendelian randomization study of inflammatory bowel disease and bone mineral density. BMC Med. 2020;18:312. 10.1186/s12916-020-01778-5 PMC7654011 33167994

[24] Burgess S Thompson SG. Interpreting findings from Mendelian randomization using the MR-Egger method. Eur J Epidemiol. 2017;32:377–89. 10.1007/s10654-017-0255-x PMC5506233 28527048

[25] Bowden J Smith GD Haycock PC Burgess S. Consistent estimation in mendelian randomization with some invalid instruments using a weighted median estimator. Genet Epidemiol. 2016;40:304–14. 10.1002/gepi.21965 PMC4849733 27061298

[26] Carter AR Sanderson E Hammerton G. Mendelian randomisation for mediation analysis: current methods and challenges for implementation. Eur J Epidemiol. 2021;36:465–78. 10.1007/s10654-021-00757-1 PMC8159796 33961203

[27] Bouras E Karhunen V Gill D. Circulating inflammatory cytokines and risk of five cancers: a Mendelian randomization analysis. BMC Med. 2022;20:3. 10.1186/s12916-021-02193-0 PMC8750876 35012533

[28] Simo M Root JC Vaquero L. Cognitive and brain structural changes in a lung cancer population. J Thorac Oncol. 2015;10:38–45. 10.1097/JTO.0000000000000345 PMC5657249 25325778

[29] Devinsky O Morrell MJ Vogt BA. Contributions of anterior cingulate cortex to behaviour. Brain. 1995;118 (Pt 1)(Pt 1):279–306. 10.1093/brain/118.1.279 7895011

[30] Whitton AE Webb CA Dillon DG. Pretreatment rostral anterior cingulate cortex connectivity with salience network predicts depression recovery: findings from the EMBARC randomized clinical trial. Biol Psychiatry. 2019;85:872–80. 10.1016/j.biopsych.2018.12.007 PMC6499696 30718038

[31] Mossinger H Kostev K. Depression is associated with an increased risk of subsequent cancer diagnosis: a retrospective cohort study with 235,404 patients. Brain Sci. 2023;13:302. 10.3390/brainsci13020302 PMC9954234 36831845

[32] Yang X Yang G Wang R. Brain glucose metabolism on [18F]-FDG PET/CT: a dynamic biomarker predicting depression and anxiety in cancer patients. Front Oncol. 2023;13:1098943. 10.3389/fonc.2023.1098943 PMC10248443 37305568

[33] Hopwood P Stephens RJ. Depression in patients with lung cancer: prevalence and risk factors derived from quality-of-life data. J Clin Oncol. 2000;18:893–903. 10.1200/JCO.2000.18.4.893 10673533

[34] Schoenberg PLA Song AK Mohr EM Rogers BP Peterson TE Murphy BA. Increased microglia activation in late non-central nervous system cancer survivors links to chronic systemic symptomatology. Hum Brain Mapp. 2023;44:6001–19. 10.1002/hbm.26491 PMC10619383 37751068

[35] McDonald BC. Structural neuroimaging findings related to adult non-CNS cancer and treatment: review, integration, and implications for treatment of cognitive dysfunction. Neurotherapeutics. 2021;18:792–810. 10.1007/s13311-021-01096-5 PMC8423886 34402034

[36] Foundas AL Leonard CM Gilmore RL Fennell EB Heilman KM. Pars triangularis asymmetry and language dominance. Proc Natl Acad Sci USA. 1996;93:719–22. 10.1073/pnas.93.2.719 PMC40120 8570622

[37] Satoh H Tanaka K Ohara G Ishikawa H Ohtsuka M Hizawa N. Cognitive impairment assessment in elderly lung cancer patients. Lung Cancer. 2007;58:429–30. 10.1016/j.lungcan.2007.08.011 17959268

[38] Dündar ZP Güçlü MB Çiltaş A Benekli M. Comparison of exercise capacity respiratory muscle strength and depression in non-small cell lung cancer patients and healthy controls. Eur Respir J. 2020;56(suppl 64):279.

[39] Smith HR. Depression in cancer patients: pathogenesis, implications and treatment (Review). Oncol Lett. 2015;9:1509–14. 10.3892/ol.2015.2944 PMC4356432 25788991

[40] Graus F Vogrig A Muniz-Castrillo S. Updated diagnostic criteria for paraneoplastic neurologic syndromes. Neurol-Neuroimmunol. 2021;8:e1014. 10.1212/NXI.0000000000001014 PMC8237398 34006622

[41] Ochenduszko S Wilk B Dabrowska J Herman-Sucharska I Dubis A Puskulluoglu M. Paraneoplastic limbic encephalitis in a patient with extensive disease small-cell lung cancer. Mol Clin Oncol. 2017;6:575–8. 10.3892/mco.2017.1162 PMC5374934 28413671

[42] Andela CD van der Werff SJA Pannekoek JN. Smaller grey matter volumes in the anterior cingulate cortex and greater cerebellar volumes in patients with long-term remission of Cushing’s disease: a case-control study. Eur J Endocrinol. 2013;169:811–9. 10.1530/EJE-13-0471 24031092

[43] Lin Y Wang S Zhang L Yang Q. Elucidating the relationship between breast cancer and brain cortical structure: a Mendelian randomization study. Cereb Cortex. 2024;34:1–8. 10.1093/cercor/bhad498 38112592

[44] Zhu Q Li Z Cai C Li D. Exploring the liver-brain axes: insights from a bidirectional Mendelian randomization study on brain cortical structure and liver cancers. Discov Oncol. 2025;16:1446. 10.1007/s12672-025-03276-7 PMC12311080 40739408

[45] Liu T Tuo X Zhao H Wang Y Jiang Y Lu J. Exploring causal relationships between brain imaging-derived phenotypes and ovarian cancer risk: a bidirectional Mendelian randomization. J Ovarian Res. 2025;18:173. 10.1186/s13048-025-01733-z PMC12315427 40751201

